# Genetic Variants and Protein Alterations of Selenium- and T-2 Toxin-Responsive Genes Are Associated With Chondrocytic Damage in Endemic Osteoarthropathy

**DOI:** 10.3389/fgene.2021.773534

**Published:** 2022-01-11

**Authors:** Yujie Ning, Minhan Hu, Jiayu Diao, Yi Gong, Ruitian Huang, Sijie Chen, Feiyu Zhang, Yanli Liu, Feihong Chen, Pan Zhang, Guanghui Zhao, Yanhai Chang, Ke Xu, Rong Zhou, Cheng Li, Feng Zhang, Mikko Lammi, Xi Wang, Xiong Guo

**Affiliations:** ^1^ Key Laboratory of Trace Elements and Endemic Diseases, School of Public Health, Health Science Center, Xi’an Jiaotong University, National Health Commission of the People’s Republic of China, Xi’an, China; ^2^ Shaanxi Provincial People’s Hospital, Xi’an, China; ^3^ Xi’an Honghui Hospital, Xi’an, China; ^4^ Shaanxi Provincial Institute for Endemic Disease Control, Xi’an, China; ^5^ Department of Integrative Medical Biology, University of Umeå, Umeå, Sweden

**Keywords:** single nucleotide polymorphism, Kashin–Beck disease, chondrocyte damage, selenium, T-2 toxin

## Abstract

The mechanism of environmental factors in Kashin–Beck disease (KBD) remains unknown. We aimed to identify single nucleotide polymorphisms (SNPs) and protein alterations of selenium- and T-2 toxin–responsive genes to provide new evidence of chondrocytic damage in KBD. This study sampled the cubital venous blood of 258 subjects including 129 sex-matched KBD patients and 129 healthy controls for SNP detection. We applied an additive model, a dominant model, and a recessive model to identify significant SNPs. We then used the Comparative Toxicogenomics Database (CTD) to select selenium- and T-2 toxin–responsive genes with the candidate SNP loci. Finally, immunohistochemistry was applied to verify the protein expression of candidate genes in knee cartilage obtained from 15 subjects including 5 KBD, 5 osteoarthritis (OA), and 5 healthy controls. Forty-nine SNPs were genotyped in the current study. The C allele of rs6494629 was less frequent in KBD than in the controls (OR = 0.63, *p* = 0.011). Based on the CTD database, PPARG, ADAM12, IL6, SMAD3, and TIMP2 were identified to interact with selenium, sodium selenite, and T-2 toxin. KBD was found to be significantly associated with rs12629751 of PPARG (additive model: OR = 0.46, *p* = 0.012; dominant model: OR = 0.45, *p* = 0.049; recessive model: OR = 0.18, *p* = 0.018), rs1871054 of ADAM12 (dominant model: OR = 2.19, *p* = 0.022), rs1800796 of IL6 (dominant model: OR = 0.30, *p* = 0.003), rs6494629 of SMAD3 (additive model: OR = 0.65, *p* = 0.019; dominant model: OR = 0.52, *p* = 0.012), and rs4789936 of TIMP2 (recessive model: OR = 5.90, *p* = 0.024). Immunohistochemistry verified significantly upregulated PPARG, ADAM12, SMAD3, and TIMP2 in KBD compared with OA and normal controls (*p* < 0.05). Genetic polymorphisms of PPARG, ADAM12, SMAD3, and TIMP2 may contribute to the risk of KBD. These genes could promote the pathogenesis of KBD by disturbing ECM homeostasis.

## Introduction

Kashin–Beck disease (KBD) is an endemic osteoarthropathy distributed throughout North Korea, Siberia, Japan, and China. In the 21st century, this condition has been most prevalent in China. Endemic areas encompass 13 provinces (or city/autonomous regions) and 379 counties from the northeast to the southwest. Monitoring data reported in the 2018 China Health Statistical Yearbook documents 535,878 patients, including 8,540 juvenile cases and more than 104 million residents at risk (http://www.nhfpc.gov.cn). Moreover, new cases have been diagnosed in Tibet recently (Ya-qun et al., 2014). The main pathogenic sites of KBD were irreversible coagulation necrosis and apoptosis in chondrocytes from articular cartilage, epiphyseal cartilage, and epiphyseal plate cartilage. Selenium deficiency and T-2 toxin (Guo et al., 2014; Sun et al., 2019; Wang et al., 2020) have been established and widely accepted as the main environmental risk factors for KBD that induce cartilage damage such as acceleration of chondrocyte apoptosis and an imbalance of the extracellular matrix (Wang et al., 2006; Li et al., 2012). However, the role of selenium deficiency and T-2 toxin in KBD development remains unclear, which limits the effective treatment options. Osteoarthritis (OA) is another cartilage-damaging osteoarthropathy that affects people worldwide (Ji et al., 2019). The aetiology and pathogenesis of OA are also poorly understood, but OA mainly occurs in elderly individuals, with chondrocyte apoptosis starting from the superficial zone of cartilage, whereas KBD mainly occurs in childhood, with chondrocyte apoptosis starting from the deep zone of cartilage (Guo et al., 2014; He et al., 2018; Wang et al., 2021).

Single nucleotide polymorphisms (SNPs) typically indicate disease susceptibility. Recently, a number of studies have suggested that SNPs play a crucial role in revealing the pathogenesis of osteochondral diseases such as OA (den Hollander et al., 2018) and KBD (Zhang et al., 2016). For example, ITPR2 has been identified as a susceptibility gene for KBD in both Han and Tibetan Chinese individuals (Ehret, 2010; Zhang et al., 2015). Shi et al. (2011) found that genetic variants in the HLA-DRB1 gene significantly increased susceptibility to KBD. Recently, TP63 was also identified as a novel susceptibility gene for KBD (Cheng et al., 2021). Advanced KBD is similar to osteoarthritis in clinical manifestations, such as arthritic pain, morning stiffness, and the deformity of limb joints. However, few studies have compared the differences in SNPs between OA and KBD, which could be helpful in genetically distinguishing the two diseases. In addition, accumulating evidence suggests that cartilage damage in patients with KBD is driven by the interaction between genetic and environmental factors (Guo et al., 2014). However, no previous study has provided any insight into SNPs in environmentally responsive genes before.

In this study, we selected 49 SNPs of 41 genes to perform SNP genotyping in 129 KBD and 129 normal controls. A Sequenom MassARRAY®SNP analysis was used to detect the associations between KBD and the 49 candidate SNPs. Immunohistochemistry was used to verify the distribution and protein expression of five candidate genes that interacted with selenium, sodium selenite, and T-2 toxin. Our results identify potential SNP biomarkers of selenium deficiency– and T-2 toxin–responsive genes to help reveal the pathogenesis of KBD.

## Methods and Materials

### Ethics

All subjects provided informed consent (orally, if the subject was illiterate) for sample collection. The study protocol was approved by the ethics committee of Xi’an Jiaotong University (Approval No. 2018-206).

### Subjects and Sample Collection

Diagnosis and degree classification of juvenile and adult KBD patients were strictly applied according to the national criteria of KBD in China [WS/T 207-2010]. Patients with OA were strictly diagnosed according to the Modified Outerbridge Classification. All subjects were diagnosed with KBD if they manifested X-ray alterations such as defects and sclerosis on the bone end of phalanges combined with compression changes of the calcaneus and talus and enlarged/deformed limb joints (hand, elbow, knee, ankle, etc.). KBD subjects were excluded if they were suffering or had previously suffered from any other osteoarticular diseases such as osteoarthritis, rheumatoid arthritis, gout, or skeletal fluorosis. Patients with any type of macrosomia, disorder of osteochondrodysplasia, chronic disease (such as hypertension, diabetes, or a coronary heart disease), or autoimmune disease were excluded if they had accepted any treatments that could affect the skeletal system within the last 6 months. All healthy controls lacked musculoskeletal pathologies or recent injuries and were selected from the same endemic areas as the KBD cases. All subjects were of Chinese Han lineage from Tongde and Guide Counties in Qinghai Province. The subjects were summoned to the local CDC and township health center for sample collection. Using the inclusion and exclusion criteria described above, we selected 129 patients with KBD and 129 healthy controls for this study. The articular cartilage samples from KBD and OA patients were collected from individuals who underwent arthroplasty of the knee. Healthy controls were obtained from patients who had suffered trauma or amputation due to an accident.

For SNP detection, approximately 5–7 ml of venous blood was collected from 258 subjects (129 KBD and 129 normal). The samples were rested for 5–10 min. First, the samples were centrifuged at 1,500×g for 5 min, and the supernatant was collected. The samples were then centrifuged at 12,000×g for 10 min, and the supernatant was collected. The resulting serum was then frozen in liquid nitrogen overnight and preserved at −80°C until testing. None of the samples was thawed more than twice before being analyzed.

Because the incidence of KBD has decreased dramatically and there are almost no new juvenile cases, it is extremely difficult to collect juvenile samples for verification. Moreover, the adult samples used here were typical KBD cases that were strictly screened. In addition, the OA patients were all adults. For these reasons, we chose only adult KBD patients for immunohistochemistry (IHC) verification. We included 15 adult subjects (5 KBD patients, 5 OA patients, and 5 normal controls) who had undergone arthroplasty of the knee or had died due to accidents or other diseases (not related to osteochondrosis) in Shaanxi Province to verify the protein expression of candidate genes in articular cartilage using immunohistochemistry. The knee joint samples were collected with the informed consent of patients and their families.

### Selection of Candidate SNP Loci

**FIGURE 1:**
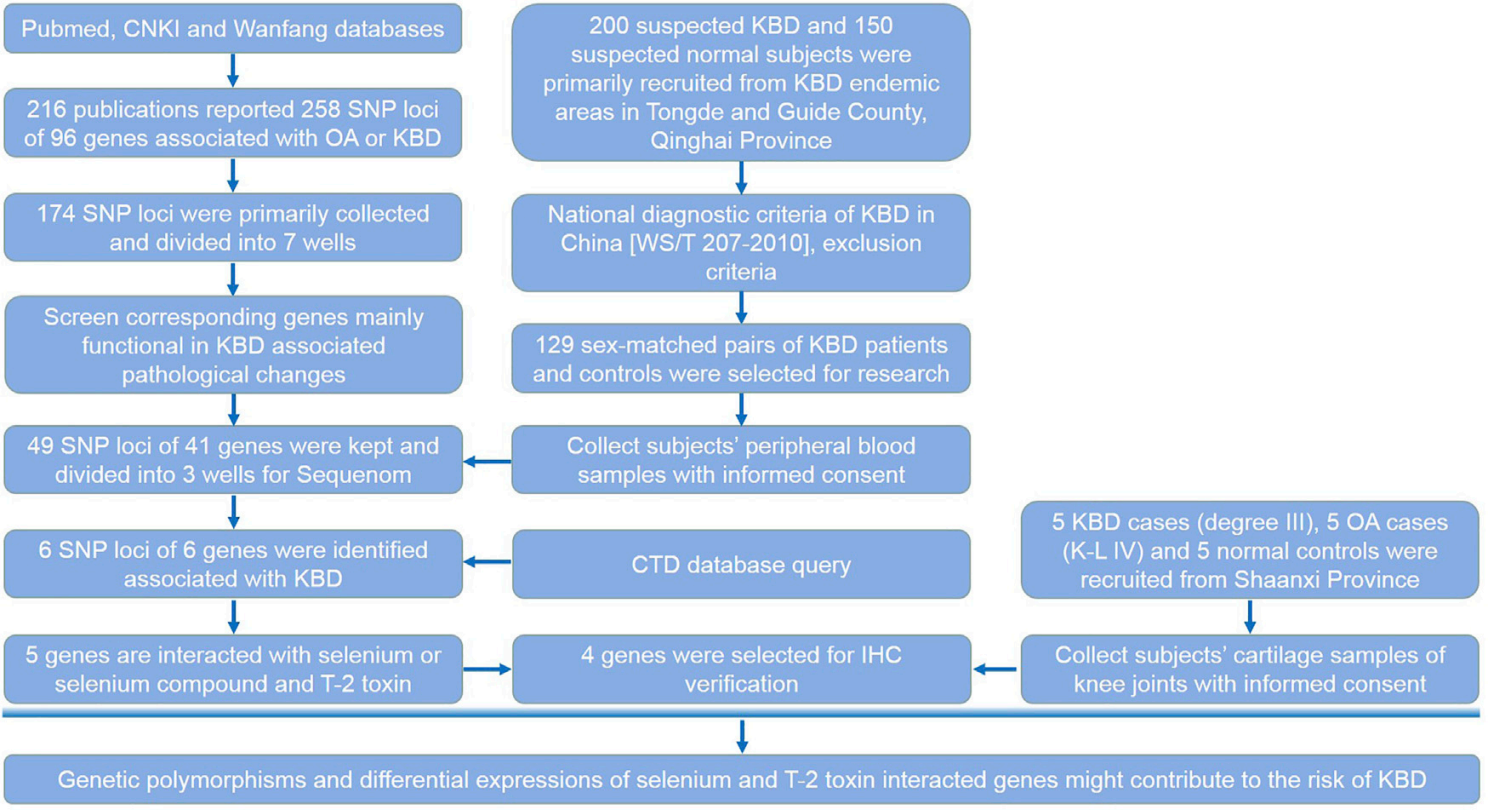
Research design. This flow chart shows the selection process of candidate SNP loci and corresponding genes, subjects recruited and sample collection procedures, and the main methods and technologies used in this study.

We determined the SNP loci reported in the literatures (Supplementary Table S1). For unpublished results, we determined the fold change value of the corresponding genes tested in KBD chondrocytes or monocytes by microarray analysis. Up to the date of the experiment, 216 publications have reported SNP loci associated with OA or KBD; these were searched from the Pubmed, CNKI, and Wanfang databases. Candidate loci also met the following criteria: the Hardy–Weinberg law (PHWE >0.05), a minimal allele frequency (MAF) > 0.05, and an R^2^ > 0.8 (default value). First, 258 SNP loci of 96 corresponding genes were collected from previous publications. Of these, 49 SNP loci of 41 corresponding genes that were differentially expressed in KBD or OA articular cartilage and/or peripheral blood mononuclear cells were selected for verification. These genes were mainly functional in KBD-associated pathological changes such as apoptosis, extracellular matrix metabolism, aggrecan, collagen, selenoprotein synthesis, and signal transduction. The selection process of candidate SNP loci and their corresponding genes is shown in detail in the research design (Figure 1).

### Primer Design

PCR amplification primers and single-base extension primers for the tested SNP loci were designed using Sequenom’s (United States) Genotyping Tools and MassARRAY Assay Design software and submitted to Biotech for synthesis.

### DNA Extraction

DNA was extracted from blood samples using a Wizard Genomic DNA Purification Kit (Promega, United States). DNA quantification and quality testing were determined by spectrophotometry and agarose gel electrophoresis, respectively. Qualified DNA was adjusted to 50 ng/μl, transferred to a 384-well plate, and stored at −20°C.

### SNP Detection

Sequenom MassARRAY SNP detection was applied to reflect the base difference caused by SNP polymorphisms using the difference in molecular weight. Using matrix-assisted laser desorption ionization time-of-flight mass spectrometry (MALDI-TOF-MS), the molecular weight of the elongated product was determined and processed using MassArray TYPER 4.0 software. The analysis was conducted in cooperation with CapitalBio Corporation (Beijing, China).

### Interactions Between Selenium, T-2 Toxin, and Genes Corresponding to SNP Loci

The Comparative Toxicogenomics Database (CTD, http://ctdbase.org/) provides manually curated information about chemical–gene/protein interactions and chemical–disease and gene–disease relationships mainly based on publications. These data are integrated with functional and pathway data to assist in developing hypotheses regarding the mechanisms of environmentally influenced diseases. This database was used to explore whether selenium, sodium selenite, and/or T-2 toxin interact with a corresponding gene to a candidate SNP locus.

### Cartilage Tissue Collection and Immunohistochemistry Verification

We identified six genes with differential SNPs: TLR3, IL6, PPARG, ADAM12, SMAD3, and TIMP2. Of these, IL6 had already been verified in the cartilage tissue or cells of KBD (Zhou et al., 2014) and OA patients (Ansari et al., 2019). However, the PPARG, ADAM12, SMAD3, and TIMP2 genes have either not been compared/verified with respect to KBD/OA, and the research conclusions are inconsistent. In addition, compared with the CTD database, no interaction was found between the TLR3 gene and T-2 toxin/low selenium, whereas PPARG, ADAM12, SMAD3, and TIMP2 interacted with both selenium and T-2 toxin. Therefore, we chose to verify the protein levels of these four genes: PPARG, ADAM12, SMAD3, and TIMP2.

All articular cartilage samples, including all of the cartilage zones (including the calcified zone) and the subchondral bone, were harvested from the lateral tibial plateau 1 h after operation. Cartilage tissues were fixed with 4% (w/v) paraformaldehyde for 24 h immediately after acquisition and decalcified in 10% (w/v) ethylenediaminetetraacetic acid disodium salt (·EDTA-2Na) for 2–3 weeks. The samples were dehydrated in an alcohol series, cleared in xylene, and embedded in paraffin wax. Paraffin sections were cut into 5-µm sections, mounted on slides, and stored at room temperature until use for staining. The paraffin-embedded sections were baked at 65°C for 1 h, deparaffinized with xylene, and then rehydrated in decreasing concentrations of ethanol. Endogenous peroxidase activity was blocked with 0.3% (w/v) hydrogen peroxide for 10 min at room temperature, and then the sections were washed with 1×PBS. The sections were then incubated in a 10 mol/L urea solution diluted with water at 37°C for 20 min and washed with 1×PBS. The sections were then treated with 2 mg/ml hyaluronidase at pH 5.0 and 37°C for 30 min. Blocking with 5% (w/v) goat serum for 20 min at room temperature was followed by the addition of anti-PPARG (1:100; Abcam, United Kingdom), anti-ADAM12 (1:100; Abcam, United Kingdom), anti-SMAD3 (1:100; Abcam, United Kingdom), and anti-TIMP2 (1:100; Abcam, United Kingdom) primary antibodies overnight at 4°C with IgG as a negative control. The slides were removed and held at room temperature for 30 min and then washed three times with PBS. Next, secondary antibodies were added at 37°C for 20 min. After washing, SABC reagent (Zhongshan Jinqiao, China) was added, and the slides were incubated at 37°C for 20 min. Finally, DAB (Abcam) staining, haematoxylin slight counterstaining, and neutral balsam fixation were performed. Instead of the primary antibody, PBS was used in each experiment as a negative control. Chondrocytes with brown granules in the nucleus and the cytoplasm were considered to be positive for the target protein.

### Statistical Analysis

For the SNP polymorphisms of candidate genes in KBD, PLINK 1.90 was used to perform an association analysis based on an additive model, a dominant model, and a recessive model to identify significant SNPs associated with the disease. Specifically, for an SNP at a certain locus, whether a genotype increased the risk of disease was considered. For example, in the GT mutation, G is less prevalent and T is prevalent. When GG, GT, and TT differ in diseases, we designated this as an additive model (i.e., if there is a G or T, it will increase the possibility of onset). The analysis of (GG + GT) vs. TT represents the dominant model of G. The analysis of GG vs. (TT + GT) represents the recessive model of G. In most cases, the less prevalent nucleotide is the disease onset SNP, which invokes the referred dominant model or recessive model. The results of each model can be divided into two parts, adjusted covariates and unadjusted covariates, depending on the presence of uncorrected confounding factors, such as age and sex.

For immunohistochemistry, a German semiquantitative scoring system was used to evaluate the staining. The staining intensity and area extent were evaluated separately with a 0 for no staining, one for weak staining, two for moderate staining, and three for strong staining. In addition, the percentage of staining was given a score of 0 (<5%), 1 (5–25%), 2 (25–50%), 3 (51–75%), or 4 (>75%). These two scores were multiplied to obtain the final score for comparison.

SPSS18.0 was used for the comparative analyses. A Student’s *t*-test was applied to determine the difference in continuous variables between two groups. A chi-square test was applied to determine the difference in categorical variables between two groups. A *p* value <0.05 indicates a statistically significant difference.

## Results

### Characteristics of the Population

The population included for SNP analysis was sex-matched. The composition of males and females between the KBD and control groups was not significantly different (*p* = 0.081). There were more juveniles (*n* = 90) than adults (*n* = 39) in the KBD group, whereas the opposite situation existed in the control group (juveniles = 3 and adults = 126; *p* < 0.001, Table 1). Table 2 shows the general information of the 15 subjects for the immunohistochemistry analysis. Five subjects were separately included in the KBD, OA, and normal control groups.

**TABLE 1:** General information of subjects for SNP detection.

		KBD (*n* = 129)	Normal (*n* = 129)	χ^2^	*p*
Sex	Male	71	57	3.039	0.081
	Female	58	72		
Age groups	Juvenile (≤18 years)	90	3	127.260	1.63E-29
	Adult (>18 years)	39	126		

Notes: *p* < 0.05 denotes significant difference. KBD, is short for Kashin-Beck disease.

**TABLE 2:** General information of subjects for IHC verification.

Number	KBD			OA			NC	
	Sex	Age	Degree	Sex	Age	K-L score	Sex	Age
1	Female	63	III	Female	69	IV	Male	37
2	Male	56	III	Male	76	IV	Male	70
3	Male	50	III	Female	81	IV	Female	62
4	Female	58	III	Male	55	IV	Male	40
5	Male	57	III	Male	63	IV	Male	56

Notes: All cartilage samples were harvested from the lateral tibial plateau of knee joints of subjects. KBD, is short for Kashin-Beck disease; OA, is short for osteoarthritis; NC, is short for normal control. Such abbreviations mean the same in the following tables and figures.

### Association Analyses of Candidate SNP Loci

Forty-nine SNPs with qualifing Hardy–Weinberg test results (Supplementary Table S2) were genotyped in the current study. In our sample, allele C of rs6494629 was less frequent in cases than in controls (OR = 0.63, *p* = 0.011, Table 3). SNP rs12629751 of PPARG was significantly associated with KBD under the additive (OR = 0.46, *p* = 0.012), dominant (OR = 0.45, *p* = 0.049), and recessive (OR = 0.18, *p* = 0.018) models after adjustment for age and sex. SNP rs6494629 of SMAD3 was significantly associated with KBD under the additive (OR = 0.65, *p* = 0.019) and dominant (OR = 0.52, *p* = 0.012) models. SNP rs3775296 of TLR-3 (OR = 0.60, *p* = 0.045) was significantly associated with KBD under the dominant model. SNP rs1800796 of IL6 (OR = 0.30, *p* = 0.003) and SNP rs1871054 of ADAM12 (OR = 2.19, *p* = 0.022) were significantly associated with KBD under the dominant model before and after adjustment for age and sex. SNP rs4789936 of TIMP2 (OR = 5.90, *p* = 0.024) was significantly associated with KBD under the recessive model after adjustment for age and sex (Table 4).

**TABLE 3:** Comparison of genotypes and alleles between the KBD and control groups.

Chromosome/SNP	Genotype/allele	KBD (*n*, %)	Control (*n*, %)	χ^2^	*p* value
3/rs12629751 T/C	TT	5 (3.91)	12 (9.52)	3.336	0.189
	TC	47 (36.72)	41 (32.54)		
	CC	76 (59.37)	73 (57.94)		
	T	57 (22.27)	65 (25.79)	0.866	0.352
	C	199 (77.73)	187 (74.21)		
4/rs3775296 A/C	AA	9 (6.98)	6 (4.76)	6.191	0.045
	AC	39 (30.23)	57 (45.24)		
	CC	81 (62.79)	63 (0.50)		
	A	57 (22.09)	69 (27.38)	1.916	0.166
	C	201 (77.91)	183 (72.62)		
7/rs1800796 G/C	GG	8 (6.20)	23 (18.11)	8.958	0.011
	GC	60 (46.51)	47 (37.01)		
	CC	61 (47.29)	57 (44.88)		
	G	76 (29.46)	93 (36.61)	2.965	0.085
	C	182 (70.54)	161 (63.39)		
10/rs1871054 T/C	TT	29 (22.48)	15 (11.81)	5.223	0.073
	TC	62 (48.06)	67 (52.76)		
	CC	38 (29.46)	45 (35.43)		
	T	120 (46.51)	97 (38.19)	3.63	0.057
	C	138 (53.49)	157 (61.81)		
15/rs6494629 C/T	CC	15 (11.72)	23 (17.97)	6.847	0.033
	CT	52 (40.63)	64 (0.50)		
	TT	61 (47.65)	41 (32.03)		
	C	82 (32.03)	110 (42.97)	6.533	0.011
	T	174 (67.97)	146 (57.03)		
17/rs4789936 T/C	TT	11 (8.59)	9 (7.03)	0.398	0.819
	TC	45 (35.16)	49 (38.28)		
	CC	72 (56.25)	70 (54.69)		
	T	67 (26.17)	67 (26.17)	0	1
	C	189 (73.83)	189 (73.83)		

Notes: *p* < 0.05 denotes significant difference. SNP, is short for single nucleotide polymorphism, and such abbreviation means the same in the following tables and figures.

**TABLE 4:** Candidate SNP loci with KBD identified by association analysis.

Chromosome/SNP	Model	OR (95%CI)	*p* value	Adjusted OR (95% CI)	Adjusted *p* value
3/rs12629751 T/C	Additive	0.78 (0.52, 1.17)	0.226	0.46 (0.25, 0.85)	0.012
	Dominant	0.93 (0.56, 1.53)	0.775	0.45 (0.20, 0.99)	0.049
	Recessive	0.38 (0.13, 1.12)	0.071	0.18 (0.05, 0.75)	0.018
4/rs3775296 A/C	Additive	0.77 (0.51, 1.17)	0.226	0.76 (0.42, 1.40)	0.382
	Dominant	0.60 (0.36, 0.99)	0.045	0.67 (0.32, 1.38)	0.272
	Recessive	1.51 (0.52, 4.38)	0.443	1.16 (0.20, 6.63)	0.870
7/rs1800796 G/C	Additive	0.75 (0.52, 1.08)	0.120	0.78 (0.46, 1.33)	0.359
	Dominant	0.92 (0.56, 1.51)	0.750	1.04 (0.50, 2.16)	0.909
	Recessive	0.30 (0.13, 0.70)	0.003	0.31 (0.10, 0.97)	0.043
10/rs1871054 T/C	Additive	1.38 (0.96, 1.99)	0.085	1.54 (0.92, 2.59)	0.102
	Dominant	1.30 (0.77, 2.20)	0.328	1.32 (0.62, 2.81)	0.475
	Recessive	2.19 (1.11, 4.31)	0.022	2.89 (1.08, 7.75)	0.035
15/rs6494629 C/T	Additive	0.65 (0.45, 0.93)	0.019	0.84 (0.48, 1.46)	0.541
	Dominant	0.52 (0.31, 0.87)	0.012	0.71 (0.34, 1.50)	0.371
	Recessive	0.60 (0.30, 1.21)	0.152	1.05 (0.35, 3.15)	0.934
17/rs4789936 T/C	Additive	1.03 (0.70, 1.52)	0.879	1.31 (0.73, 2.36)	0.368
	Dominant	0.96 (0.58, 1.57)	0.856	1.00 (0.48, 2.06)	0.998
	Recessive	1.40 (0.54, 3.60)	0.485	5.90 (1.26, 27.59)	0.024

Notes: Data have been adjusted by age and sex; *p* < 0.05 denotes significant difference.

### Interactions Between Environmental Risk Factors and Responsive Genes With Candidate SNPs

Based on the CTD, five candidate genes (PPARG, ADAM12, IL6, SMAD3, and TIMP2) were found to interact with selenium, sodium selenite, and T-2 toxin. These chemicals could affect the expression of the above genes at both the gene and protein levels to participate in KBD-associated pathogenic processes such as apoptosis, changes to the extracellular matrix, and ROS reactions. Details of the interactions are summarized in Table 5.

**TABLE 5:** Candidate SNP loci corresponding genes and associated interactions with selenium and T-2 toxin.

SNP loci	Gene name	Associated chemicals	Interactions from the CTD database	References: Pubmed ID
rs12629751	PPARG	Selenium	PPARG protein promotes the reaction [Selenium inhibits the reaction [lipopolysaccharide, *Escherichia coli* O111 B4 results in increased expression of PTGS2 protein]]	17439952
			Selenium promotes the reaction [lipopolysaccharide, *Escherichia coli* O111 B4 results in increased activity of PPARG protein]	17439952
		Sodium selenite	Sodium selenite inhibits the reaction [2-cresol results in increased expression of PPARG mRNA]	21705299
			Sodium selenite results in increased expression of PPARG protein	24140496
		T-2 toxin	T-2 toxin results in increased expression of PPARG mRNA	26141394
rs1800796	IL6	Selenium	[Cadmium analog co-treated with selenium analog co-treated with zinc sulfide analog] results in increased expression of IL6 mRNA	21481475
			Selenium affects the reaction [mercuric chloride results in decreased expression of IL6 protein]	26089086
		Sodium selenite	[Sodium selenite co-treated with plant extracts] inhibits the reaction [sodium arsenite results in increased expression of IL6 protein]	26085057
			Sodium selenite inhibits the reaction [cadmium chloride results in increased expression of IL6 protein]	24954678
			Sodium selenite inhibits the reaction [IL6 protein results in increased activity of AR protein]	17346688
			Sodium selenite inhibits the reaction [IL6 protein results in increased expression of KLK3 protein]	17346688
			Sodium selenite inhibits the reaction [sodium arsenite results in increased expression of IL6 protein]	26085057
			Sodium selenite inhibits the reaction [TGFB1 protein results in increased expression of IL6 protein]	16757516
			Sodium selenite results in increased expression of IL6 mRNA	18175754
		T-2 toxin	AKNA protein affects the reaction [T-2 toxin results in increased expression of IL6 mRNA]	29079362
			Alpha-cyano-(3,4-dihydroxy)-N-benzylcinnamide inhibits the reaction [T-2 toxin results in increased expression of IL6 mRNA]	22454431
			Stattic inhibits the reaction [T-2 toxin results in increased expression of IL6 mRNA]	22454431
			T-2 toxin results in increased expression of IL6 mRNA	29079362
rs1871054	ADAM12	Sodium selenite	Sodium selenite results in decreased expression of ADAM12 mRNA	18175754
rs6494629	SMAD3	Sodium selenite	Sodium selenite results in increased expression of SMAD3 mRNA	18175754
rs4789936	TIMP2	Selenium	Selenium inhibits the reaction [T-2 toxin results in decreased expression of TIMP2 protein]	21144892
			Selenium results in decreased expression of TIMP2 mRNA	17390030
		T-2 toxin	Selenium inhibits the reaction [T-2 toxin results in decreased expression of TIMP2 protein]	21144892
			T-2 toxin affects the expression of TIMP2 mRNA	21112371
			T-2 toxin results in decreased expression of TIMP2 protein	21144892

Note: CTD, is short for Comparative Toxicogenomics Database.

### Immunohistochemistry Verification of Candidate Genes

**FIGURE 2:**
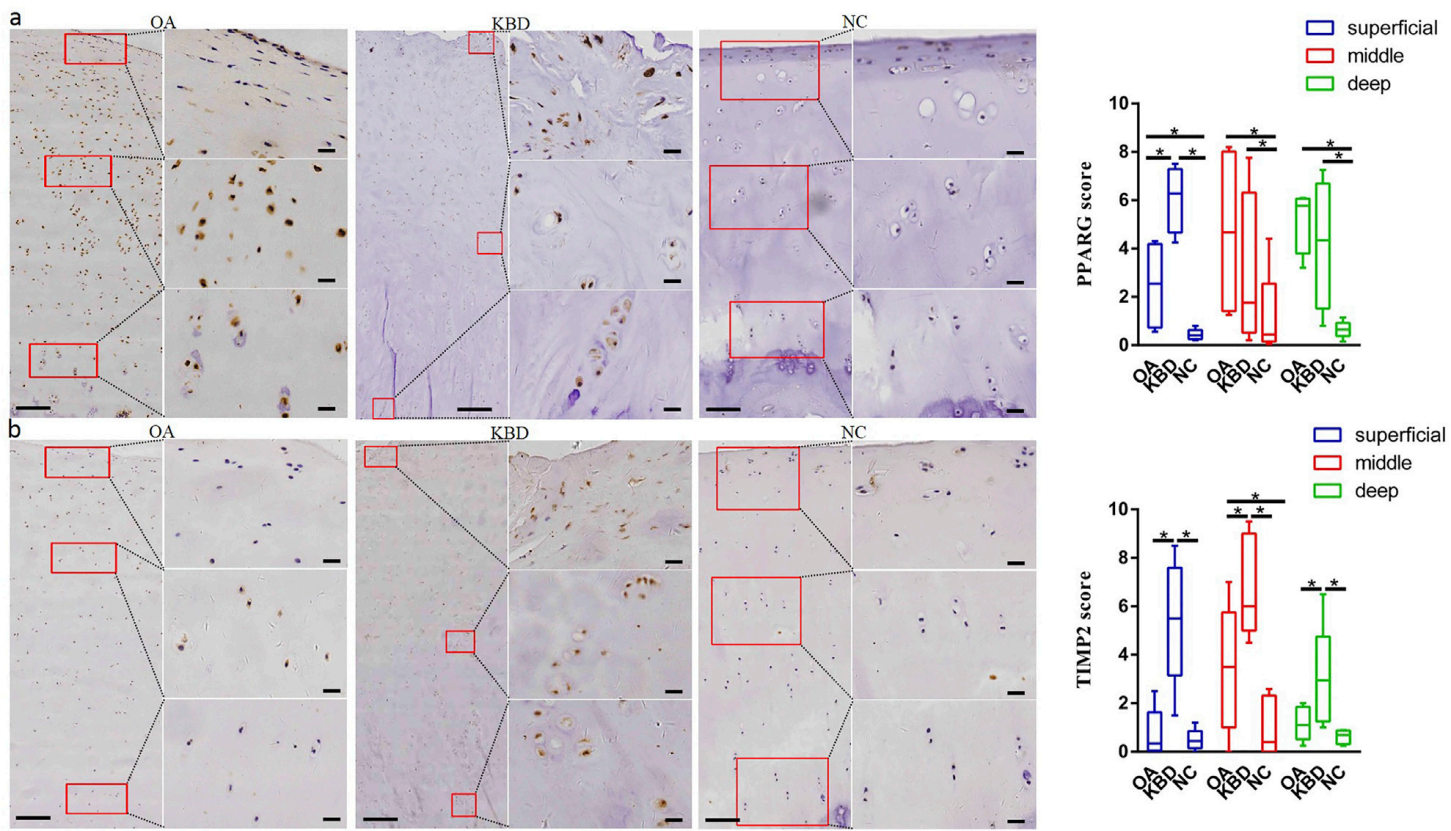
Representative immunohistochemistry staining of PPARG **(A)** and TIMP2 **(B)** in OA, KBD, and normal control cartilage tissues. The scores of different areas (superficial, middle, and deep) in cartilage tissues displayed by box plot (*n* = 5). *p < 0.05.

**FIGURE 3:**
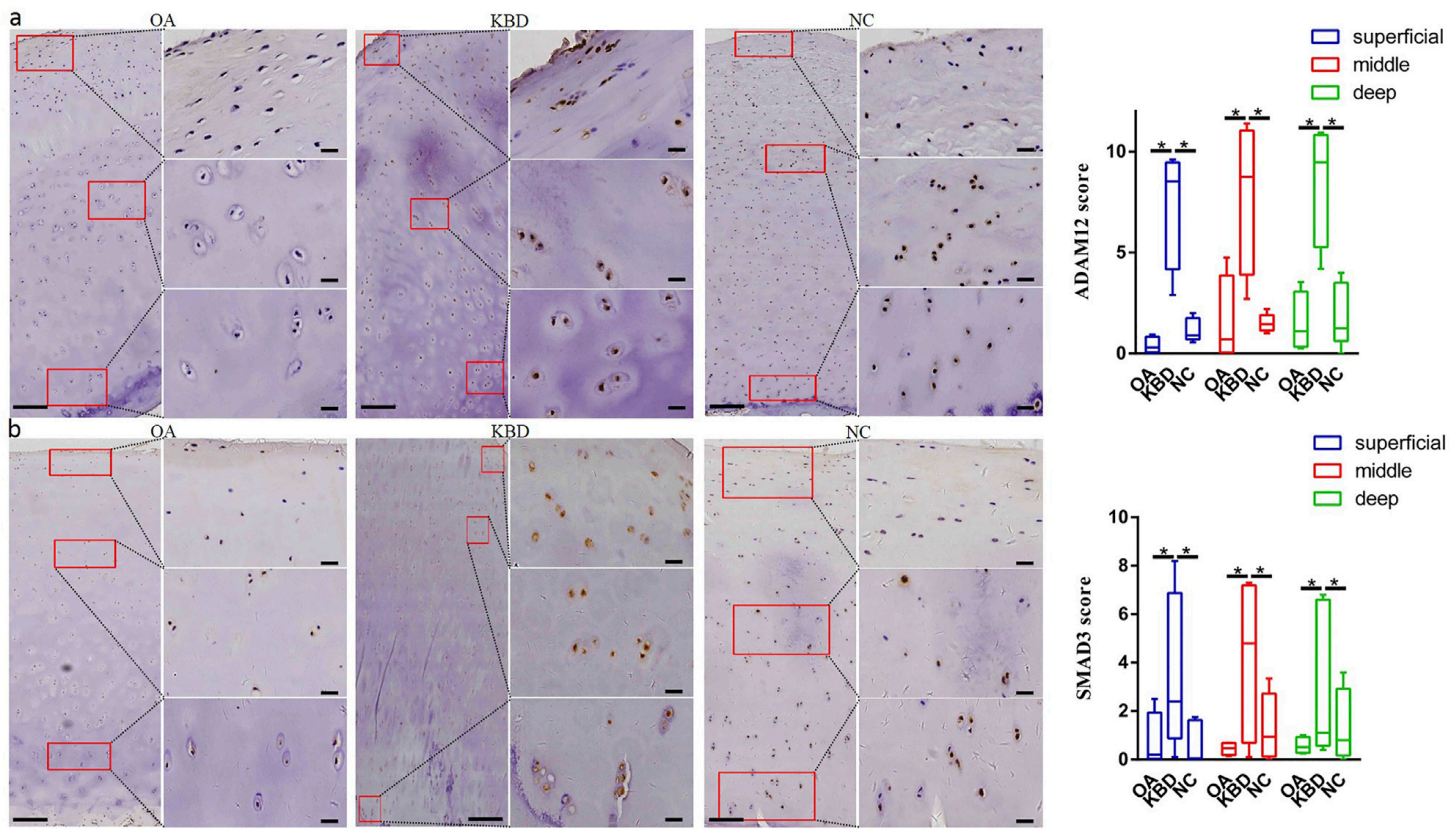
Representative immunohistochemistry staining of ADAM12 **(A)** and SMAD3 **(B)** in OA, KBD, and normal control cartilage tissues. The scores of different areas (superficial, middle, and deep) in cartilage tissues displayed by box plot (*n* = 5). *p < 0.05.

Immunohistochemistry was used to verify the distribution and protein expression of PPARG, ADAM12, SMAD3, and TIMP2 in adult articular cartilage obtained from KBD and OA patients and normal controls. The gene PPARG was barely expressed in normal controls but significantly upregulated in KBD, particularly in the superficial zone and the deep zone (Figure 2A, *p* = 0.016, *p* = 0.034). This protein was also significantly upregulated in OA compared with the controls (*p* = 0.041) and showed slight but insignificant upregulation in the middle and deep zones compared with KBD. TIMP2 was significantly upregulated in all zones of KBD compared with OA and the normal control and upregulated in the middle zone of OA compared with the normal control (Figure 2B, *p* = 0.026). The genes ADAM12 (Figure 3A, *p* = 0.002) and SMAD3 (Figure 3B, *p* = 0.036) were significantly upregulated in all zones of KBD compared with OA and the normal control.

## Discussion

Gene–environment interactions have been widely used in studying the aetiology and pathogenesis of complex diseases (Li et al., 2019) and have been demonstrated to be associated with KBD and OA (He et al., 2021). Gene polymorphisms may participate in the development of KBD (Wu et al., 2019) and OA (Styrkarsdottir et al., 2018). Our study recruited 49 candidate SNPs from publications on osteochondropathy. KBD was found to be significantly associated with SNPs rs12629751 T/C of PPARG on chromosome 3, rs3775296 A/C of TLR-3 on chromosome 4, rs1800796 G/C of IL6 on chromosome 7, rs1871054 T/C of ADAM12 on chromosome 10, rs6494629 C/T of SMAD3 on chromosome 15, and rs4789936 T/C of TIMP2 on chromosome 17. These responsive genes (PPARG, ADAM12, SMAD3, and TIMP2) were selected for verification based on the interactions with environmental risk factors for KBD such as low selenium and T-2 toxin. The candidate genes were verified mainly upregulated in articular cartilage in KBD compared with those in OA patients and controls. Therefore, SNP and protein alterations of these genes could participate in the pathogenic process of KBD.

The gene PPARG (peroxisome proliferator activated receptor gamma), also known as PPARγ, is involved in cell apoptosis and necessary in cartilage differentiation (Monemdjou et al., 2012). However, PPARG plays a double-edged role in cartilage. The PPARG polymorphism rs12629751 was identified to be significantly associated with susceptibility to knee OA in a southeast Chinese population (Ding et al., 2014). Overexpression of miR-27b inhibited the expression of PPARG, matrix metalloproteinase 13 (Mmp13), and type X collagen (Col10a1), while significantly promoting the expression of type II collagen (Col2a1) and sex-determining region-box 9 (Sox9) at both the mRNA and protein levels. However, agonist- and siRNA-mediated knockdown of PPARG suppressed Col2a1 expression while promoting the expression of Col10a1 and runt-related transcription factor 2 (Runx2) in a concentration-dependent manner (Xu et al., 2018). Aberrant alterations in these genes and proteins are the main molecular characteristics of KBD cartilage injury (Wang et al., 2008; Wang et al., 2017). One of our unpublished results demonstrated that exosomal hsa-miR-27b-3p was significantly downregulated in KBD chondrocytes (FC = 0.53, *p* = 0.0176). Therefore, the upregulation of PPARG might contribute to KBD cartilage damage. Moreover, the treatment of KBD by injecting hyaluronic acid into the knee joint is effective in alleviating arthritic pain and morning stiffness, hence promoting patient quality of life (Yu et al., 2014; Xia et al., 2016; Tang et al., 2019). Sodium hyaluronate could protect articular cartilage against degeneration by inhibiting PPARG mRNA expression (Zhou et al., 2009). This could represent a partial therapeutic mechanism of hyaluronic acid injection for KBD therapy.

The gene ADAM12 (ADAM metallopeptidase domain 12) encodes a member of the matrix metalloproteinases and is involved in skeletal growth and development (Kveiborg et al., 2008). It is capable of stimulating longitudinal bone growth by modulating chondrocyte proliferation and maturation (Kveiborg et al., 2006). However, a meta-analysis including 5,048 cases and 6,848 controls suggested that rs1871054 was significantly associated with the risk of knee OA (Hu et al., 2017). In addition, upregulation of ADAM12 may participate in abnormal chondrocyte differentiation and accelerate OA development (Yang et al., 2017). The gene ADAM12 was expressed in 87% of OA cartilages at both the mRNA and protein levels compared with normal controls (Okada et al., 2008). ADAM12 was upregulated prior to Col10a1 during chondrogenic differentiation in ATDC5 cells. In addition, TGF-β1–induced ADAM12 overexpression resulted in upregulation of Igf-1 and downregulation of Runx2 expression (Horita et al., 2019). The protein RUNX2 is a crucial transcription factor for type X collagen expression and chondrocyte hypertrophy and is known to regulate endochondral ossification through the control of chondrocyte proliferation and differentiation (Chen et al., 2014). Abnormal endochondral ossification during childhood is the main pathogenesis of KBD. Downregulation of COL2A1 and RUNX2 and upregulation of COL10A1 are consistently observed in KBD chondrocytes (Guo et al., 2006; Wang et al., 2008). Therefore, upregulated ADAM12 could be an accelerating factor for KBD development.

SMAD3 (SMAD Family Member 3) is a protein-encoding gene. The SMAD3 protein transmits signals from the cell surface to the nucleus and functions in the TGF-β signaling pathway, which is critical in the proliferation, differentiation, migration, and apoptosis as well as extracellular matrix (ECM) synthesis and degradation (Heldin et al., 1997; Wharton and Derynck, 2009). SMAD3 polymorphisms are associated with the risk of both hip and knee arthritis. Specifically, the SNP locus rs6494629 mapping to intron one of SMAD3 was associated with knee OA (Sharma et al., 2018). Thus, abnormal SMAD3 expression was expected in damaged cartilage. Previously, SMAD3 expression was reported to be, on average, 83% higher in OA cartilage than that in controls (Aref-Eshghi et al., 2016). Indeed, the SMAD3 protein was significantly overexpressed in KBD and OA cartilages in our study. The TGF-β1/SMAD3 signaling pathway has been reported to be essential for the inhibition of chondrocyte differentiation. Interestingly, T-2 toxin reduced the expression of type II collagen while promoting the expression of MMP13 through the activation of SMAD3 and the stimulation of TGF-β1 signaling, which ultimately led to chondrocyte damage. Chondrocytes undergo abnormal terminal differentiation, which is another pathogenic characteristic of KBD (Li et al., 2017).

The gene TIMP2 (tissue inhibitor of matrix metalloproteinase 2) maintains extracellular balance. The SNP rs4789936 on TIMP-2 was observed to decrease the risk of alcohol-induced osteonecrosis of the femoral head (ONFH) in the Chinese Han population under allele, dominant, overdominant, and log-additive models after adjusting for age and sex (Chen et al., 2016). Choi et al. found that hyperactivation of SMAD3 signaling during the osteogenic differentiation of Costello syndrome (CS) MSCs leads to aberrant expression of ECM remodeling proteins such as MMP13, TIMP1, and TIMP2. Specifically, enhanced TIMP1/2 expression induced by hyperactivated SMAD3 signaling impairs the osteogenic development of CS MSCs *via* inactivation of wnt/β-catenin signaling (Choi et al., 2021). Compared with normal nucleus pulposus (NP) tissues, intervertebral disc degeneration (IDD) samples exhibited higher levels of circular RNA derived from TIMP2 (circ-TIMP2) expression levels. In addition, overexpression of circ-TIMP2 promoted ECM catabolism and suppressed ECM anabolism. Furthermore, circ-TIMP2 sequesters miR-185-5p, which potentially upregulates the target genes associated with ECM degradation (Guo et al., 2020). Therefore, TIMP2 imbalance could disturb ECM homeostasis in KBD cartilage.

The results were obtained based on a literature review, Sequenom MassARRAY SNP detection, CTD database query, and IHC verification. However, due to the dramatically decreased incidence of KBD in recent years, the sample size for SNP analysis is relatively small, which indicates that we may have missed other SNP loci indicative of susceptibility to KBD as affected by low selenium and T-2 toxin.

## Conclusion

The evidence suggests that SNPs and upregulated expression of low selenium– and T-2 toxin–responsive genes, including PPARG, ADAM12, SMAD3, and TIMP2, could participate in the pathogenesis of KBD by disturbing ECM homeostasis. The functions of these genes appear to be linked through the TGF-β and wnt/β-catenin pathways, which needs further investigation.

## Data Availability

The datasets presented in this study can be found in online repositories. The names of the repository/repositories and accession number(s) can be found in the article/https://www.frontiersin.org/articles/10.3389/fgene.2021.773534/full# Supplementary Material.
